# FollowNet: A Comprehensive Benchmark for Car-Following Behavior Modeling

**DOI:** 10.1038/s41597-023-02718-7

**Published:** 2023-11-25

**Authors:** Xianda Chen, Meixin Zhu, Kehua Chen, Pengqin Wang, Hongliang Lu, Hui Zhong, Xu Han, Xuesong Wang, Yinhai Wang

**Affiliations:** 1 Systems Hub, Intelligent Transportation Thrust, The Hong Kong University of Science and Technology (Guangzhou), Guangzhou, 511400 China; 2 Guangdong Provincial Key Lab of Integrated Communication, Sensing and Computation for Ubiquitous Internet of Things, Guangzhou, 511400 China; 3 Department of Civil and Environmental Engineering, The Hong Kong University of Science and Technology, Hong Kong, Hong Kong; 4 Information Hub, Data Science and Analytics Thrust, The Hong Kong University of Science and Technology (Guangzhou), Guangzhou, 511400 China; 5 School of Transportation Engineering, Tongji University, Shanghai, 201804 China; 6 Department of Civil and Environmental Engineering, University of Washington, Seattle, 98195 Washington USA

**Keywords:** Civil engineering, Computer science

## Abstract

Car-following is a control process in which a following vehicle adjusts its acceleration to keep a safe distance from the lead vehicle. Recently, there has been a booming of data-driven models that enable more accurate modeling of car-following through real-world driving datasets. Although there are several public datasets available, their formats are not always consistent, making it challenging to determine the state-of-the-art models and how well a new model performs compared to existing ones. To address this gap and promote the development of microscopic traffic flow modeling, we establish the first public benchmark dataset for car-following behavior modeling. This benchmark consists of more than 80 K car-following events extracted from five public driving datasets under the same criteria. To give an overview of current progress in car-following modeling, we implemented and tested representative baseline models within the benchmark. The established benchmark provides researchers with consistent data formats and metrics for cross-comparing different car-following models, coming with open datasets and codes.

## Introduction

Car-following is the most fundamental and frequent driving behavior. It involves actions taken by a driver when following another vehicle ahead. Proper car-following behavior reduces crashes and improves traffic flow stability^1–4^. The corresponding car-following model is a mathematical or computational representation of the behavior exhibited by drivers when following other vehicles on the road. It describes and predicts the dynamics of following vehicles (FV) and lead vehicles (LV) movements in traffic flow and serves as a cornerstone for microscopic traffic simulation^5–7^.

Over the past decade, there has been a boom in data-driven car-following models, primarily due to the availability of real-world driving data and advancements in machine learning. Representative data-driven car-following models include neural network based^8,9^, recurrent neural network based^10,11^, and reinforcement learning based^12–15^. However, existing research has several limitations. Firstly, there is a lack of standardized data formats and evaluation criteria for car-following models. Although several public driving datasets are available, such as NGSIM and HighD, there are no uniform data formats and evaluation criteria for car-following models, making it challenging to compare newly proposed models with existing ones in terms of performance. Secondly, current research inadequately represents car-following behavior in mixed traffic flows due to limited datasets. As we enter a transitional phase where self-driving and human-driven vehicles share the road, previous studies have mainly focused on modeling car-following behavior using limited datasets that do not account for autonomous vehicles.

**Fig. 1:**
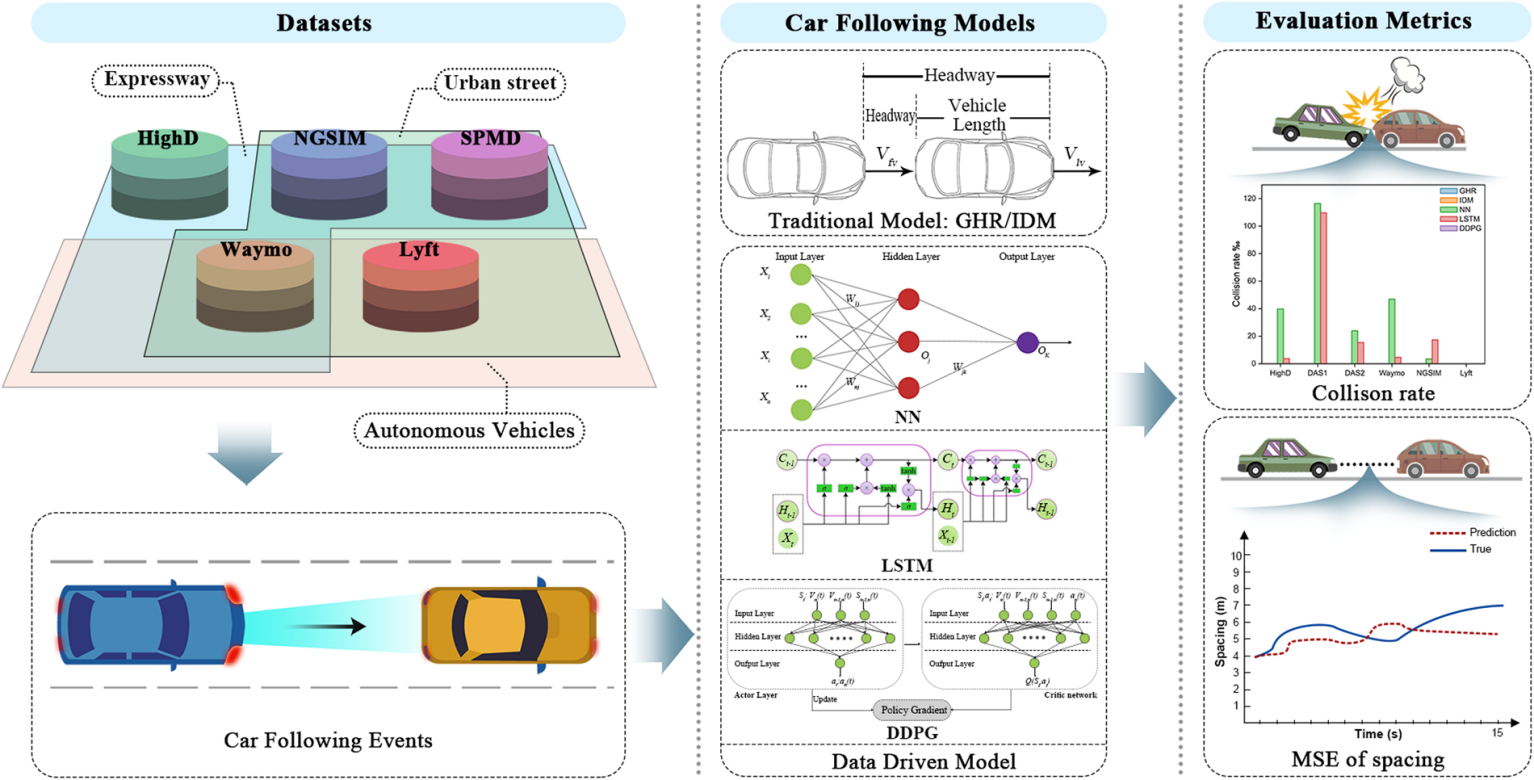
A road map of car-following benchmark: FollowNet.

To further advance the field of microscopic traffic simulation modeling, it is imperative to establish a public car-following benchmark that can address the aforementioned issues and serve as a standard dataset. Similar to how standard datasets such as ImageNet^16^, Microsoft COCO^17^, and KITTI^18^ have contributed to their respective fields, a car-following benchmark would be beneficial for advancing the field of microscopic traffic flow modeling. To achieve this, we developed a benchmark called FollowNet. The benchmark was created by extracting car-following events from five publicly available datasets using consistent criteria. Within the benchmark, we implemented and tested five baseline car-following models, including both traditional and data-driven approaches (Fig. 1). This paper summarizes recent developments in this field, as well as evaluates the performance of mainstream models using our benchmark.

Our study has the following contributions:

- We systematically summarized existing car-following models.
- We established the first benchmark for car-following behavior with consistent data formats to streamline the development of car-following models. Our standardized car-following benchmark addresses the challenges of handling various data structures and frameworks from different datasets.
- We provided various scenarios, including mixed traffic and different road types, to test representative car-following models under standardized metrics.
- Our open-sourced data and codebase provided valuable insights for car-following data extraction, model calibration, and baseline implementation.

## Related work

### Traditional car-following models

Since the Pipes model^19^ was proposed in 1953, researchers have studied car-following models for 70 years. In general, existing traditional car-following models include five main categories: stimulus-response model, safe distance model, psycho-physiological model, optimal velocity model, and desired goal model^20–22^.

The stimulus-response model uses the relative distance or relative speed to determine the acceleration of the FV. The Gazis-Herman-Rothery (GHR) model^23^, the first stimulus-response model, assumes that a vehicle’s acceleration positively relates to the speed difference and that the FV’s response is expressed regarding its acceleration or deceleration behavior. The basic equation of this model can be expressed as:

1 $${a}_{n}(t)=c{v}_{n}^{m}(t)\frac{\Delta v(t-T)}{\Delta {x}^{l}(t-T)}$$

where the *n*-th vehicle’s acceleration at time *t* is represented by *a*_*n*_(*t*), Δ*v*, and Δ*x* are the speed difference and distance between the (*n−*1)-th vehicle and the *n*-th vehicle, respectively. *m*, *c*, and *l* are constants that need to be calculated, and *T* is the driver’s reaction time. The General Motors (GM) model^24^ is another well-known stimulus-response model that uses the difference of the speed and space headway to determine the acceleration.

Kometani *et al*.^25^ propose the first safety distance model. The underlying principle of the model differs from the stimulus-response model due to the LV’s unpredictable motion. This model is also known as the conflict avoidance model since the FV always keeps a minimum safe distance. The basic formula is:

2 $$\Delta x(t-T)=\alpha {v}_{n-1}^{2}(t-T)+{\beta }_{l}{v}_{n}^{2}(t)+\beta {v}_{n}(t)+{b}_{0}$$

where Δ*x* denotes the distance between the (*n*−1)-th vehicle and the *n*-th vehicle, *v*_*n*_(*t*) is the speed of the *n*-th vehicle at time *t*, *T* is the driver’s reaction time, *α*, *β*_*l*_, *β*, *b*_0_ are parameters to be calibrated. Similarly, Gipps model^5^ utilized the concept of the safe distance to facilitate car-following behavior, the driver of the FV selects a certain speed and maintains a corresponding distance from the LV to prevent a collision.

The psycho-physiological model suggests that drivers adopt strategies based on the relative motion between the LV and FV, including changes in speed and distance differences, and only react when the threshold value is exceeded. This type of model was first proposed by Michaels^26^, and is used in VISSIM®, a microscopic traffic simulation tool, through the Wiedemann model^27^.

The optimal velocity model (OVM) is introduced by Bando *et al*.^28^, which can account for various traffic flow phenomena, such as free flow, congested traffic, the relationship between density and traffic flow, and stop-and-go traffic waves. However, the OVM model may produce unrealistic acceleration and deceleration processes. To address this limitation, the generalized force (GF) model^29^ is proposed, which adds the effect of negative speed difference to the optimal velocity model. However, both models neglect situations where the speed of the LV is much slower than that of the FV. The full velocity difference (FVD) model^30^ considers the safety distance and offers a more precise acceleration function, which is given by:

3 $${a}_{n}(t)=\alpha [{V}_{n}^{\ast }(\Delta {X}_{n}(t))-{V}_{n}(t)]+\lambda (\Delta {V}_{n}(t))$$

4 $$\lambda =\{\begin{array}{cc}{\lambda }_{0}: & \Delta {X}_{n}(t)\le {s}_{c}\\ 0: & \Delta {X}_{n}(t) > {s}_{c}\end{array}$$

The first term in the acceleration function is proportional to the difference between the optimal velocity ${V}_{n}^{\ast }(\Delta {X}_{n}(t))$ and the actual velocity *V*_*n*_(*t*), and the second term considers the velocity difference Δ*V*_*n*_(*t*) as a linear stimulus. The sensitivity coefficients are denoted by *α* and *λ*, and *s*_*c*_ is a threshold value that distinguishes between following and free driving.

The desired goal model assumes that each driver has certain desired goals to achieve, such as desired following speed, desired headway, etc. The Intelligent Driver Model (IDM)^31^ is recognized as the most commonly employed driver-based desired goal model. It stands out as a comprehensive and concise theoretical model that prioritizes safety and accident prevention. According to IDM, each driver aims to maintain a unique set of desired parameter values during car-following behavior. The model expressions are:

5 $${a}_{n}(t)={a}_{0}\left[1-{\left(\frac{{v}_{n}(t)}{{\widetilde{v}}_{n}}\right)}^{\lambda }-{\left(\frac{{\widetilde{S}}_{n}(t)}{{s}_{n}}\right)}^{2}\right]$$

6 $${\widetilde{S}}_{n}(t)={S}_{0}+{v}_{n}(t)\widetilde{T}+\frac{{v}_{n}(t)\Delta v(t)}{2\sqrt{{a}_{0}b}}$$

7 $$\Delta v(t)={v}_{n}(t)-{v}_{n-1}(t)$$

where *a*_*n*_(*t*) and *v*_*n*_(*t*) represent the acceleration and the velocity of the FV at time *t*, respectively. And *S*_*n*_(*t*), Δ*v*(*t*) are the spacing and relative speed between the FV and the LV. The desired maximum acceleration, comfortable deceleration, desired velocity, and desired time headway are represented by *a*_0_, *b*, $\widetilde{v}$, and $\widetilde{T}$, respectively. *S*_0_ is the minimum safe headway and *λ* is a constant to be calibrated. Subsequently, the Intelligent Driver Model with Memory (IDMM)^32^ is introduced which redesigns the IDM to incorporate memory effects and adapt driving behavior to the surrounding traffic. Furthermore, the human driver model^33^ is applied to the IDM, which incorporates an advanced anticipative and smooth braking strategy, achieving impressive performance on real traffic data. Also, the IDM is well-designed with stochasticity to study various traffic flow oscillations, including string instability, external white acceleration noise, and indifference regions^34^.

Traditional car-following models may have several potential drawbacks, such as making oversimplified assumptions about driver behavior and neglecting individual driving differences or diverse driving styles. Despite these limitations, traditional ones still hold value in the study of traffic engineering and flow analysis^35^. For example, the IDM can cope with any traffic situation on cities, rural roads, or freeways with human-driven and autonomous leaders, and it has even been successfully applied to bicycle traffic flow and served as the basis for lane-free mixed traffic flow in developing countries such as India^36^. More advanced car-following models have been developed that are able to get around some of these limitations due to recent advancements in computing as well as data collection techniques.

### Data-driven car-following models

**Fig. 2:**
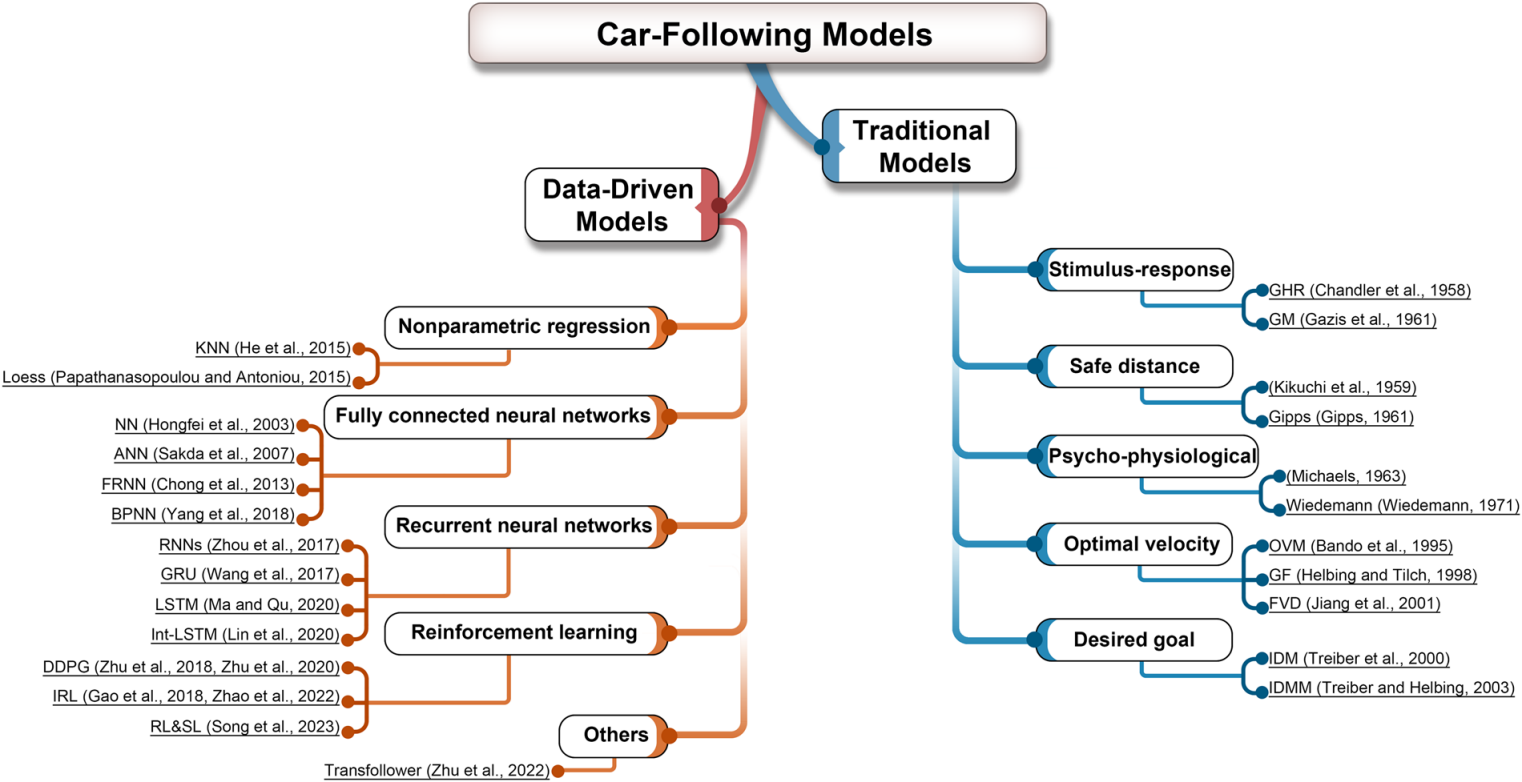
The development of car-following models.

Traditional models and data-driven models are the two main categories of car-following models, as shown in Fig. 2. Data-driven models make use of artificial intelligence techniques such as nonparametric regression, fully connected neural networks, recurrent neural networks, reinforcement learning, and other methods to predict drivers’ behavior. These models learn relationships between different factors and the driver’s behavior from the collected data.

A straightforward k-nearest neighbor (KNN) based nonparametric car-following model^37^ is presented in 2015 that forecasts the most likely driving behavior under the given circumstances. Similarly, Loess model^38^ is also a nonparametric data-driven car-following model based on locally weighted regression.

Another common approach to predict the acceleration of the FV is to use a fully connected neural network (NN). A 4-layer neural network with two hidden layers^39^ takes the gap distance, the relative velocity, desired velocity, and FV’s speed as inputs to predict the FV’s acceleration directly. The reactive agent-based car-following models are proposed by Panwai *et al*.^40^ using artificial neural network (ANN) techniques. The results show that the models’ degrees of accuracy are best for the backpropagation architecture, which is a widely used neural network algorithm for supervised learning, and the fuzzy ARTMAP architecture, which is a type of neural network that combines fuzzy logic and adaptive resonance theory. Also, one hidden layer neural network can accurately predict acceleration and Chong *et al*.^9^ comes up with the fuzzy rule-based neural network (FRNN), which is further improved by giving the neural network an instantaneous reaction time (RT) delay^41^. In addition, a Back-Propagation Neural Networks (BPNN) model^42^ combines with the Gipps model to avoid crashes.

Recurrent neural networks are another popular network structure that can model temporal problems such as vehicle following using historical information. Vanilla RNN^10^ is utilized to simulate car-following behavior and demonstrates effective traffic oscillation prediction. Two other popular RNN variants, Long Short-Term Memory (LSTM) and Gated Recurrent Unit (GRU) have also been used to model car-following behavior. Ma *et al*.^43^ utilize LSTM to address issues of gradient vanishing and exploding during long sequence training. Wang *et al*.^44^ use GRU which can improve training efficiency to simulate car-following behavior. To address the issue of error propagation, Lin *et al*.^45^ propose the LSTM-based interconnected car-following model (Int-LSTM).

In a reinforcement learning approach, the agent learns an optimal control policy through trial and error in an unknown environment based on a reward function. Zhu *et al*.^12^ present a framework for modeling car-following based on deep deterministic policy gradient (DDPG), which aims to accurately reproduce human-like car-following behavior. However, human driving may not be the optimal driving operation^46^, and it can be optimized in terms of safety, efficiency, and comfort^47^ rather than simply imitating human drivers. Therefore, Zhu *et al*.^13^ propose a reward function that aims to achieve two objectives: mimicking human drivers and optimizing driving performance. Driving characteristics and human driving data are used to design the reward function and the agents are trained to learn the decision mechanism by continuously utilizing history information. Also, Hart *et al*.^47^ propose and validate a novel car-following model based on modularized reinforcement learning. The model is trained using 100% artificial data and incorporates a multi-component reward function with weighted parameters that reflect driving behavior, which can partially address the black-box nature of conventional neural network models. It effectively updates speed and position using the Euler and ballistic methods^48^ and outperforms other machine learning or traditional models. Other studies^49–51^ assess individual driving characteristics and car-following behaviors using inverse reinforcement learning (IRL) or the combination of RL and Supervised Learning (SL).

In addition, a long-sequence car-following trajectory prediction model based on the Transformer attention-based model is proposed by Zhu *et al*.^52^, which follows a typical encoder-decoder architecture. The encoder uses multi-head self-attention to create a mixed representation of the past driving environment utilizing historical spacing and speed data as inputs. This model can effectively capture complex temporal relationships between the data, allowing it to produce a more accurate representation of the driving behavior.

Data-driven approaches can capture the intricate correlations among variables leading to a better representation of the car-following behavior. However, their interpretability and generalizability remain a challenge. Also, standardized testing datasets and evaluation criteria are required to compare performance and determine the best model. While many public datasets are available for evaluation purposes, their data formats and standards may differ, and significant effort is required to familiarize the data structure of each dataset and extract car-following events. Therefore, creating a car-following benchmark among public datasets can simplify the model testing process and promote the development of microscopic traffic flow modeling.

## Results

We train and test two traditional and three data-driven car-following models, namely GHR, IDM, NN, LSTM, and DDPG, within the benchmark. We first present the datasets and provide a brief overview of essential concepts for subsequent analyses. We then analyze basic statistics and patterns of car-following behavior in different datasets. Finally, we evaluate the performances of baseline models using consistent metrics.

### Dataset descriptions

The proposed benchmark consists of car-following events extracted from five mainstream public driving datasets: HgihD^53^, Next Generation Simulation (NGSIM)^54^, Safety Pilot Model Deployment (SPMD)^55^, Waymo^56^, and Lyft^57^. Specifically, car-following events in Waymo and Lyft datasets involve mixed traffic conditions. We excluded events with more than 90% of time being still. Depending on the characteristics of each dataset, additional filtering rules may also be applied to smooth the data, as presented later in this paper. Table 1 presents basic meta information for these datasets.

**Table 1:** Five driving datasets covered by FollowNet.

Dataset	Viewpoint	Scene	Sensors	AV involved	Data Hz
HighD	External	Expressway	Camera	No	25
NGSIM	External	Expressway, Urban street	Camera	No	10
SPMD	External	Expressway, Urban street	Camera, GPS	No	10
Waymo	Driver	Expressway, Urban street	Camera, Lidar	Yes	10
Lyft	Driver	Urban street	Camera, Lidar	Yes	10

**HighD** dataset^53,58^ is released by the Institute of Automotive Engineering at RWTH Aachen University in Germany. It is a comprehensive driving dataset that provides high-precision information about vehicle positions and speeds. The dataset includes bird’s-eye view videos of six different roads around Cologne, Germany, captured by a high-resolution 4 K camera mounted on the drone. The vehicle position and speed information are extracted using advanced computer vision techniques, which ensures that the positioning error is under 10 cm.

**NGSIM** (Next Generation Simulation)^54^ is a traffic dataset created by the Federal Highway Administration (FHWA) to study the dynamics of traffic flow on expressways. The trajectory data is collected by simultaneous photography from cameras set up on the high ground. Among them, the I-80 section is located before the evening peak between 4:00 p.m. and 4:15 p.m., and the traffic condition is relatively smooth. Car-following events of I-80 are collected based on the rebuilt data^59^ because there are measurement errors in the original data.

The **SPMD** (Safety Pilot Model Deployment) was conducted in 2014 by the U.S. Department of Transportation (USDOT) to evaluate a dedicated short-range communication technology for vehicle-to-vehicle safety applications^55^. The dataset includes basic safety messages, vehicle trajectory, driver-vehicle interaction data, and weather data. Most of the data presented in this dataset was gathered from vehicles that have both vehicle-to-infrastructure and vehicle-to-vehicle communication devices installed, in addition to several roadside sensors. The data acquisition system (DAS) is used to extract position and speed information from two driving datasets, namely DAS1 and DAS2. Since the spacing data can be obtained not only from sensors directly, but also theoretically inferred from the relative speed simulation of two consecutive frames, some noisy data can be filtered.

**Waymo**, a self-driving car company operated by Alphabet, the parent company of Google, released the Waymo Open Dataset^56^ in August 2019. It includes high-resolution sensor data from lidar and cameras, along with precise 3D vehicle poses and annotations of object information. The dataset contains various driving scenarios, such as expressways and urban streets, and has a total of 1950 scenes, each lasting 20 seconds. Hu *et al*.^60^ manually extracted car-following events based on video data of this dataset. On their basis, a total of 1440 following events were extracted.

**Lyft**^57^ is a Level 5 autonomous driving dataset, which includes a high-definition spatial semantic map, over 55,000 3D artificial annotation frames, and data collected from 7 cameras and 3 LiDARs. This dataset is particularly useful for understanding car-following behavior in mixed traffic flows as it includes more than 170,000 scenarios of human-driven vehicles following autonomous vehicles and autonomous vehicles following human-driven vehicles. Each scene is recorded for approximately 25 seconds.

### Descriptive statistics and distributions of behavioral measures

**Fig. 3:**
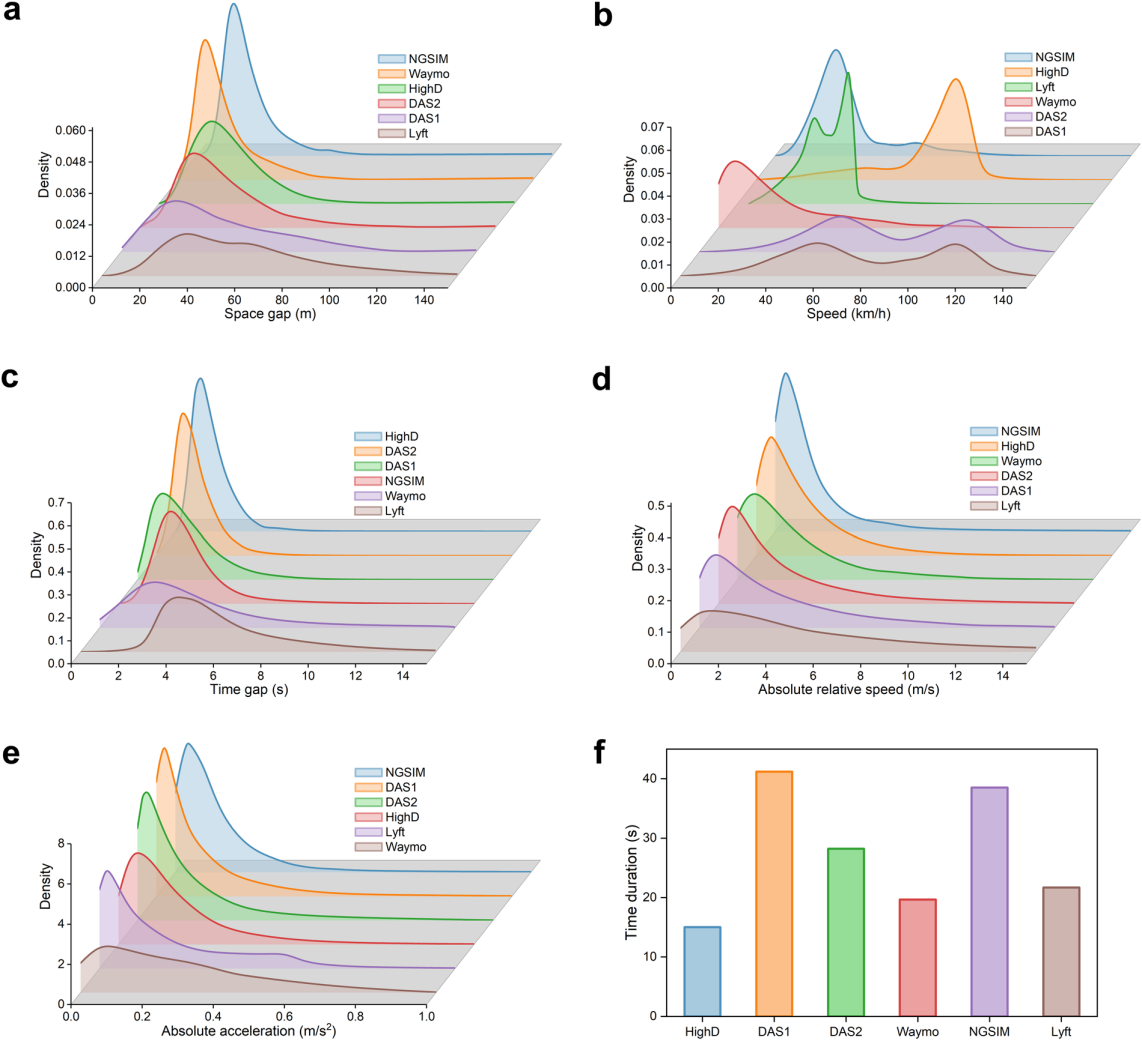
Distributions of car-following behavioral measurements: (**a**) Average space gap during car following (*m*), (**b**) Average speed during car following (*km*/*h*), (**c**) Average time gap during car following (*s*), (**d**) Average absolute relative speed during car following (*m*/*s*), (**e**) Average absolute acceleration during car following (*m*/*s*^2^), (**f**) Average event duration.

This section presents descriptive statistics and distributions of common car-following behavioral measurements for aforementioned datasets, as shown in Fig. 3. By normalizing the curve, the integral over the entire range of possible values is set to 1. We investigated car-following speed, spacing gap, time gap, relative speed, acceleration, and time duration across these datasets. Our analysis reveals that each dataset has unique characteristics related to its data collection location and driving scenarios. The following are key findings from the comparison of car-following behavior across different datasets.

#### Space gap

Lyft dataset has the largest average spacing gap. On the other hand, Waymo dataset has the smallest average spacing gap, showing that the autonomous vehicles in this dataset are maintaining a smaller following distance, likely due to driving in more congested urban environments. DAS1 and DAS2 datasets have similar spacing gap distributions with an average of around 25 meters. The spacing gap is important in evaluating car-following behavior and can help develop guidelines for setting safe following distances for autonomous vehicles.

#### Following speed

The DAS1 and DAS2 datasets have relatively high following speeds, with data distributions that are centered around 60 *km*/*h* and 110 *km*/*h*. The Waymo dataset, on the other hand, has the lowest following speeds, as the car-following events primarily occur in low-speed driving scenarios in urban streets. However, HighD exhibits high following speeds, with data distributions that are centered around 80 *km*/*h*, which is related to the expressway driving scenario.

#### Time gap

The Lyft dataset exhibits the largest time gap compared to other datasets. This dataset is particularly useful for studying car-following behavior in situations with longer following distances or larger time gaps between vehicles in urban streets. Researchers can analyze this dataset to gain insights into how drivers adjust their behavior during transitions between car-following and free-driving modes. On the other hand, the HighD dataset is characterized by small time gaps and a concentration of around 1 second. This dataset provides valuable support for studying car-following behavior on expressways, aiming to improve traffic flow and reduce congestion.

#### Absolute relative speed

Most datasets exhibit similar data distributions for absolute relative speed, suggesting that there may be some common driving behaviors across different locations and traffic conditions. However, the Lyft dataset features a different distribution, with most car-following events exhibiting absolute relative speeds in the range of 2 *m*/*s*.

#### Absolute acceleration

The statistics indicate a higher likelihood of observing higher acceleration rates in both the Waymo and Lyft datasets. Thus, car-following behaviors captured in these datasets can be characterized as relatively more aggressive. These two datasets can serve as valuable resources for developing and validating car-following models that prioritize safety and conservatism.

#### Car-following duration

The car-following duration in the HighD is set to 15 seconds, while the NGSIM and DAS1 have an average car-following duration exceeding 35 seconds. These long-duration events provide valuable data support for studying long-sequence car-following behavior, enabling us to explore both inter-driver heterogeneity and intra-driver heterogeneity in driving styles.

### Benchmark for car-following behavior modeling

Here, we provide a brief overview of our benchmark for car-following behavior modeling.

#### Data preprocessing

Car-following events are extracted from the five datasets using the same criteria: (1) having a continuous and unchanged LV ID, and (2) with a minimum duration of 15 seconds. A total of 1930, 16658, 24247, 1440, 24093, and 12540 car-following events were extracted from the NGSIM I-80, SPMD (DAS1), SPMD (DAS2), Waymo, Lyft, and HighD datasets, respectively. Furthermore, each dataset is randomly divided into three parts: the training set, validation set, and test set, with proportions of 70%, 15%, and 15% respectively. These sets are then kept fixed throughout the experiment. However, for traditional models like IDM and GHR, there are different naming conventions in traditional and machine learning (ML) models: ML training and validation corresponds to classical calibration while ML testing corresponds to classical validation. In the ML validation process, it is important to highlight that a complete re-learning is required whenever there is any change in the set of hyperparameters. The validation process involves training the ML model using a specific set of hyperparameters and evaluating its performance on a validation set. The hyperparameters are the tunable parameters that define the behavior and characteristics of the model.

#### Baseline models

We investigate the performances of two traditional car-following models (GHR, IDM) and three data-driven models (NN, LSTM, and DDPG) across the five datasets. These models are optimized using various methods such as genetic algorithms (GA) and hyperparameter adjustments to minimize the Mean Squared Error (MSE) of spacing and reproduce human-like driving behavior.

#### Evaluation metrics

To comprehensively assess the effectiveness of car-following models in maintaining safe, efficient, and comfortable driving behavior, it is important to establish a set of standard evaluation metrics. In this study, we propose four key metrics to evaluate the performance of car-following models:

- Mean square error of spacing: This metric measures the accuracy of a model in reproducing human driving behavior by evaluating the deviation between the modeled and observed spacing between vehicles. A lower mean square error indicates a better match to human driving behavior.
- Collision rate: This metric quantifies the ability of a model to avoid collisions by measuring the frequency of collision events. A lower collision rate indicates a safer car-following model.
- Driving comfort metric (jerk): The jerk metric evaluates the smoothness of vehicle acceleration or deceleration. It measures the rate of change of acceleration and reflects the comfort level experienced by the driver and passengers. A lower jerk value indicates a smoother and more comfortable driving experience.
- Time-To-Collision (TTC) metric: The TTC metric estimates the time it takes for a vehicle to collide with the LV based on their relative positions and velocities. By analyzing TTC values, we can assess the level of safety and potential collision risk. A higher TTC value indicates a safer car-following model.

### Model benchmark performance

We have presented the MSE of spacing, collision rate, jerk, and TTC as the standard evaluation criteria for assessing the effectiveness of car-following models in maintaining safe, efficient, comfortable driving behavior.

#### MSE of spacing

**Fig. 4:**
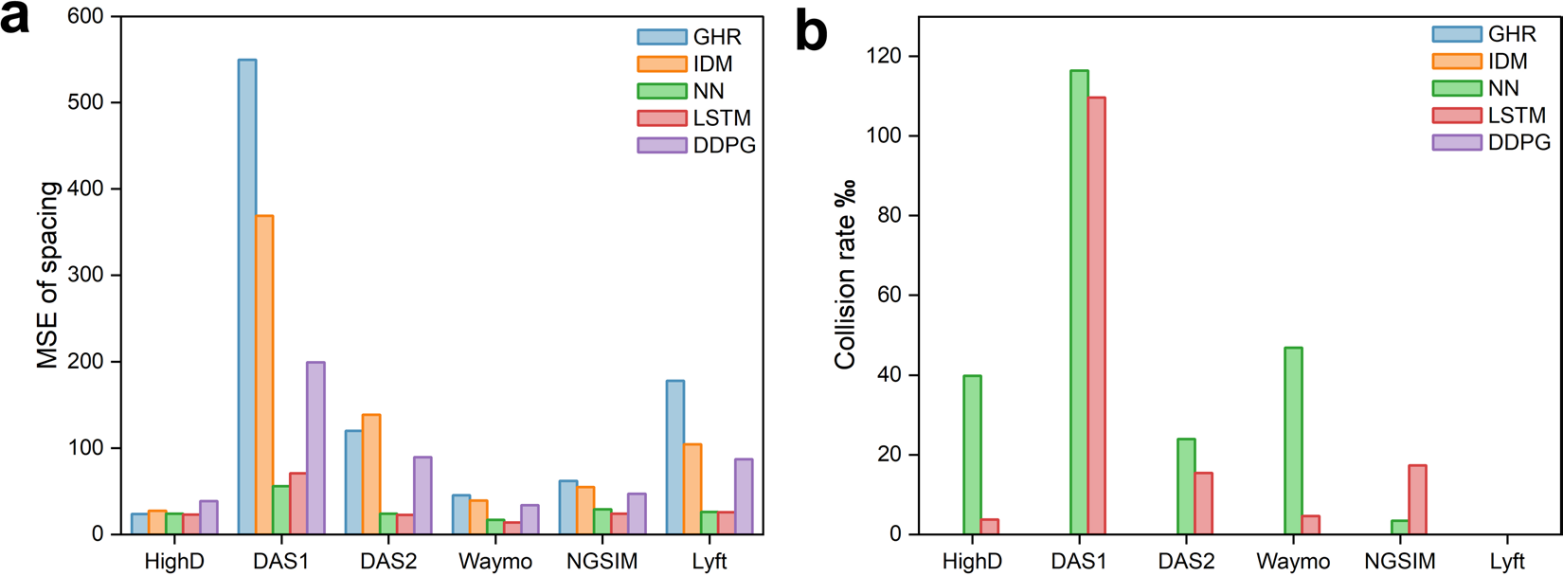
Model benchmark performance (**a**) MSE of spacing, (**b**) Collison rate.

The performance of the five car-following models, namely IDM, GHR, NN, LSTM, and DDPG, are evaluated on multiple datasets, including NGSIM, HighD, Lyft, Waymo, and SPMD. The corresponding results are presented in Table 2 and visually represented in Fig. 4. It can be concluded that data-driven models, such as NN, LSTM, and DDPG, generally exhibit lower spacing errors compared to traditional models that rely on mathematical equations except for the HighD dataset. Data-driven models can capture complex relationships between input variables, leading to more accurate predictions of car-following behavior. However, on the HighD dataset, which was collected only on expressways, GHR and IDM can still achieve competitive results in MSE spacing with zero collision. Therefore, traditional models still have their advantages in a single scenario like expressways. The research suggests that while data-driven models generally outperform traditional models, it is important to carefully consider the scenario in question when selecting the appropriate model.

**Table 2:** Test of MSE (spacing).

Model	HighD	DAS1	DAS2	Waymo	NGSIM	Lyft
GHR	23.76	549.72	119.92	45.52	62.18	178.01
IDM	27.50	368.95	138.69	39.17	54.97	104.32
NN	24.06	**56.01**	24.14	16.79	29.00	26.06
LSTM	**22.90**	70.69	**22.49**	**13.75**	**23.86**	**25.71**
DDPG	38.52	199.41	89.34	33.91	46.93	86.98

#### Collision rate

A single collision can result in severe consequences, such as injury or death. As shown in Table 3, while traditional models exhibit higher MSE of spacing, they are capable of achieving zero collision when calibrated using GA. Compared to the NN and LSTM models, the DDPG model with a carefully crafted reward function achieves zero collision in all datasets. Notably, all models demonstrate zero collision performance in the Lyft dataset. This might be attributed to the presence of large following gaps in the events captured in this dataset, as shown in Fig. 3.

**Table 3:** Collison rate‰ (Number of Collisions).

Model	HighD	DAS1	DAS2	Waymo	NGSIM	Lyft
GHR	**0**	**0**	**0**	**0**	**0**	**0**
IDM	**0**	**0**	**0**	**0**	**0**	**0**
NN	39.87 (75)	116.44 (291)	23.92 (87)	46.29 (10)	3.46 (1)	**0**
LSTM	3.72 (7)	109.64 (274)	15.39 (56)	4.62 (1)	17.30 (5)	**0**
DDPG	**0**	**0**	**0**	**0**	**0**	**0**

#### Driving comfort

Jerk measures the smoothness of acceleration changes during car-following. As shown in Table 4, LSTM demonstrates exceptional performance by achieving the lowest jerk values in the HighD, DAS2, NGSIM and Lyft datasets. This indicates that LSTM effectively minimizes abrupt changes in acceleration or deceleration, resulting in smoother car-following behavior. The NN model also performs well in the DAS1 and DAS2 datasets. Notably, IDM, being a classic car-following model, exhibited competitive performance, especially with the lowest jerk value observed in the Waymo dataset. This indicates that IDM maintains smooth acceleration and deceleration profiles, further enhancing driver comfort and ride quality. On the other hand, the DDPG model displays relatively higher jerk values across all datasets, implying a less comfortable car-following behavior.

**Table 4:** Mean absolute jerk.

Model	HighD	DAS1	DAS2	Waymo	NGSIM	Lyft
GHR	0.34	0.49	0.13	0.42	0.53	0.24
IDM	0.09	0.53	0.19	**0.22**	0.27	0.29
NN	0.08	**0.03**	**0.08**	0.32	0.75	0.15
LSTM	**0.04**	0.07	**0.08**	0.24	**0.25**	**0.14**
DDPG	33.40	8.75	23.84	7.23	8.04	31.42

#### Time-To-collision

In regards to the minimum TTC in Table 5, IDM demonstrates superior performance by achieving the highest values in the HighD, DAS2, and NGSIM datasets. These high TTC values indicate that IDM effectively maintains safe following distances between vehicles, resulting in zero collisions in all datasets. It is worth noting that LSTM achieves the highest minimum TTC value in the Lyft dataset. On the other hand, the DDPG model obtains the highest minimum TTC value in the DAS1 and Waymo datasets.

**Table 5:** Minimum of the TTC.

Model	HighD	DAS1	DAS2	Waymo	NGSIM	Lyft
GHR	101.38	31.58	85.53	61.10	14.03	78.70
IDM	**384.80**	29.69	**877.27**	40.63	**31.10**	73.71
NN	60.13	33.83	75.54	35.67	13.34	121.78
LSTM	127.68	69.51	119.77	104.37	13.74	**132.47**
DDPG	111.66	**1107.24**	32.98	**152.94**	12.18	46.47

#### Experiment on 30-second car-following behavior study

To investigate longer car-following durations in driving behavior, we extract 30-second events from the HighD dataset which has high data quality. Comparing our results to the previous 15-second experiment, we observe similar outcomes, as shown in Table 6. The LSTM model achieves the lowest MSE of spacing and jerk, while IDM and NN obtain the second-lowest jerk scores. The GHR model ranks highest in TTC. Moreover, IDM, GHR, and well-designed DDPG models achieve zero collisions, which is crucial in autonomous driving scenarios. This aspect should be emphasized and serves as an area for improvement for LSTM and NN models.

**Table 6:** Model performance comparison on long duration (30 s) car-following events.

Model	Test of MSE (spacing)	Collison rate ‰ (Number of Collisions)	Jerk	TTC
GHR	49.28	**0**	0.26	**25.05**
IDM	46.84	**0**	0.14	24.91
NN	33.38	86.29 (17)	0.14	9.91
LSTM	**31.23**	71.07 (14)	**0.13**	10.76
DDPG	53.45	**0**	22.71	22.98

## Discussion

Based on the findings mentioned above, there are several points to discuss regarding the model performance and potential future directions for car-following research.

### Safety

Although data-driven models achieve lower MSE of spacing compared to traditional models, collisions can still occur. Therefore, it is desirable to develop car-following models that not only achieve lower spacing errors but also have zero collision rates. Incorporating the ability to avoid collisions into data-driven models would significantly enhance their safety and make them suitable for real-world applications.

### Interpretability and generalization ability

Data-driven car-following models often lack interpretability and generalization ability. Each model requires specific tuning based on the dataset it is trained on. Models trained on a specific dataset, such as NGSIM, may not perform as well when directly applied to a different dataset, like Lyft. This discrepancy suggests that there are underlying variations and characteristics unique to each dataset that impact the car-following behavior. Therefore, it is necessary to develop car-following models that can generalize effectively across different datasets and driving scenarios. Understanding how well models transfer their learned knowledge between datasets can help identify limitations and guide the development of more robust and adaptable car-following models.

### Expectations for future datasets

To further enhance the performance and realism of car-following models, it is crucial to include additional factors in future datasets. For instance, incorporating data related to road conditions, and traffic signals would provide a more comprehensive understanding of the driving environment. Additionally, integrating information about surrounding vehicles and their behaviors would enable the models to account for complex interactions and make more accurate predictions. By incorporating these additional data sources, future datasets can better represent real-world driving scenarios, facilitating the development of more robust and effective car-following models.

### More advanced algorithms

Exploring advanced machine learning techniques can greatly enhance car-following models. For example, employing graph neural networks (GNNs) can help capture the intricate interactions between vehicles in a traffic network, leading to more accurate predictions of car-following behavior. Additionally, generative models can be used to synthesize new car-following events and expand the training data, enabling the models to learn from a larger and more diverse dataset. Moreover, leveraging meta-learning approaches can facilitate the development of adaptive car-following models that can quickly adapt to different datasets and driving conditions. These advanced algorithms have the potential to enhance the performance, adaptability, and scalability of car-following models, paving the way for more efficient and safe autonomous driving systems.

### Mixed traffic flow

Traffic forecasting is a critical aspect of proactive urban traffic control and management^61,62^. As we accelerate towards the era of autonomous driving, a mixed traffic landscape unfolds, where an amalgamation of autonomous and human-driven vehicles coexist on the roadways^63^. This requires developing car-following models that can effectively handle the interactions between autonomous and human-driven vehicles. Using datasets that include autonomous vehicles for training, particularly the Lyft and Waymo datasets provided in this benchmark, is crucial for the development of car-following models in mixed traffic flows.

### Driving heterogeneity

The proposed benchmark assumes homogeneous driving behavior, meaning that all vehicles follow similar patterns and behave in a consistent manner. However, in reality, driving behavior can vary significantly among different drivers, vehicle types, and traffic conditions. Therefore, incorporating driving heterogeneity into car-following models requires the development of adaptive algorithms and representative datasets that encompass different driving styles and behaviors, and traffic scenarios.

In this study, we propose FollowNet, the first benchmark of car-following behavior that includes five commonly used real-world datasets. Based on this benchmark, we have analyzed the characteristics of car-following behavior with six dimensions. Additionally, we have evaluated the performance of different car-following models, using MSE of spacing, collision rate, jerk, and TTC as evaluation metrics. The data-driven models, including NN, LSTM, and DDPG, outperform traditional models like IDM and GHR in terms of MSE of spacing. However, traditional models can achieve a zero collision rate when calibrated using GA, emphasizing the safety in car-following models is crucial. Especially, our proposed DDPGs_Max model achieves competitive performance on the benchmark with a smaller MSE of spacing than traditional models such as IDM and GHR in most datasets, as well as zero collision rate compared to NN and LSTM models. With the exception of some RL models, NN and LSTM models do not have this robustness and generalization abilities. In terms of driving comfort measured by jerk values, NN and LSTM perform exceptionally well by achieving the lowest jerk values in multiple datasets. Also, the classic IDM demonstrates competitive performance, especially with the lowest jerk value observed in the Waymo dataset. However, the DDPG model exhibits relatively higher jerk values, indicating less comfortable car-following behavior. Lastly, IDM consistently achieves higher TTC values in most datasets, indicating effective maintenance of safe following distances and zero collisions. We believe that the establishment of the car-following benchmark with open access to data and source code will enable the development of more accurate, comfortable, and safe models, ultimately contributing to the advancement of microscopic traffic simulation models.

## Methods

This section provides the design of our benchmark. First, we describe the data preprocessing and preparation steps. Second, we introduce the employed traditional models and data-driven models. Lastly, we define the evaluation metrics used in our benchmark.

### Data preparation and preprocessing

Data processing is to extract and deduce some specific valuable and meaningful data from many disordered and incomprehensible data^64,65^. The extracted car-following events utilizing a methodology similar to prior investigations^12,44,66^. Firstly, to guarantee that the FV follows the same LV throughout the event, the LV’s ID should be continuous and unchanged. Secondly, the duration of the car-following event must be 15 seconds or longer to provide sufficient data for analysis. Lastly, the lateral distance between the FV and LV must be less than 2 meters, or the FV and LV must be on the same lane, based on the characteristics of the dataset.

In order to identify the ID of the LV that was followed continuously and record the start and end time of the following event, Algorithm 1 has been applied to all datasets. To address the issue of noise in the initial data when applying datasets, we utilized the Savitzky-Golay filter for smoothing. This filter is a finite impulse response filter that employs polynomial fitting to estimate the underlying signal and mitigate the effect of noise in the time series data.

### Baseline models

In this benchmark, we trained and tested a total of five car-following models, consisting of two conventional models and three data-driven models. All models were trained with a standardized time duration of 15 seconds, with consistent input parameters including spacing, FV’s speed, and relative speed. The acceleration of the FV served as the output for all models. The five baseline models are listed as follows:

#### Algorithm 1

Find_Lead_Vehilce_ID (*x*, *data_hz*).

#### IDM

The IDM demonstrates the best predictive performance when compared with other traditional models according to Zhu *et al*.^67^. We proceeded to train this model with the objective of minimizing the MSE of spacing by employing GA with pool calibration (concatenating the calibration data part and finding a single parameter set reflecting the average driver) to determine the most effective IDM parameter set.

#### GHR

The GHR model assumes that drivers adjust their speed based on the distance to the preceding vehicle and their own desired speed. Similar to the IDM, GA was utilized to discover the optimal parameter values^67^.

#### NN

To forecast the future acceleration of the FV, a neural network model consisting of three feedforward layers is employed. The Adam optimizer with a learning rate of 0.001 is utilized for optimizing models. Following this, the MSE of spacing is utilized as the loss function for optimizing the network.

#### LSTM

The LSTM model consists of an encoder component and a linear layer. The encoder takes the input data and computes both the encoded representation and the hidden state of the final layer. Subsequently, the linear layer maps the hidden state to the output value, which undergoes a tanh activation function and is multiplied by a constant representing the acceleration limit. In order to enhance the performance of the LSTM model, several hyperparameters have been fine-tuned. These adjustments include the hidden size, the number of LSTM layers, and the utilization of a dropout probability of 0.1 to enhance the model’s resilience.

#### DDPG

**Fig. 5:**
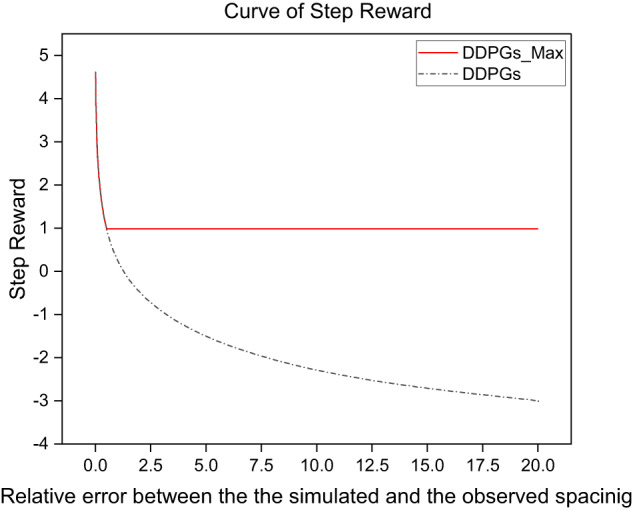
Reward function of DDPGs_Max and DDPGs.

DDPGs^12^ has been proven to be effective in minimizing the discrepancy between the simulated and actual actions in order to reproduce human-like driving. However, the proposed reward function can not be generalized to all datasets. Therefore, a modified reward function *r*_*t*_ (DDPGs_Max) with the max operation and collision penalty is proposed in this paper, which can achieve less collision and higher accuracy. It is expressed as below:

8 $$\begin{array}{c}{r}_{t}=max(-\log \left(|\frac{{S}_{n-1,n}(t)-{S}_{n-1,n}^{obs}(t)}{{S}_{n-1,n}^{obs}(t)}|\right),H)+CollisionCheck\ast Penalty\end{array}$$

where *S*_*n*-1,*n*_(*t*) and ${S}_{n-1,n}^{obs}(t)$ are the simulated and observed spacing at time step *t*, respectively. *H* is the step reward, which is an adjustable parameter, set to 1 here. The collision check with penalty is set to let the agent learn in the direction of collision reduction in the RL environment. The condition for the end of training is that a collision occurs or the exploration of the entire car-following event is completed. For a single step, the max operation borrowed from the concept of max pooling is used to ensure that the agent can complete the exploration of the entire event as much as possible. In other words, the reward comes from two aspects. Firstly, it is based on how closely the value of the single-step simulation matches the observed value. Secondly, it includes a reward for ensuring that there are no collisions during each time step of the simulation., as shown in Fig. 5.

### Evaluation metrics

Since there is no universally standardized set of evaluation metrics for assessing the effectiveness of a car-following model in maintaining safe, efficient, and comfortable driving behavior, it is essential to establish criteria that effectively capture these key aspects. For this purpose, we utilize MSE of spacing, collision rate, jerk, and TTC as evaluation metrics. A lower MSE score indicates a better alignment between the model and the data, resulting in improved accuracy when predicting the spacing between vehicles. On the other hand, high collision rates suggest that the model is ineffective in avoiding collisions. While a model with a low MSE score can accurately predict vehicle spacing, it may not excel in collision avoidance. For one car-following event, the MSE of spacing can be expressed as:

9 $${\rm{M}}{\rm{S}}{\rm{E}}=\frac{1}{N}\mathop{\sum }\limits_{i=1}^{N}{({S}_{n-1,n}(t)-{S}_{n-1,n}^{obs}(t))}^{2}$$

where *N* is the total number of observations, and *i* is an observation index.

Similarly, we define collision rate as the number of car-following events where the spacing between vehicles is less than zero divided by the total number of car-following events in the test dataset. The formula for collision rate is as follows:

10 $$Collision\,Rate=\frac{{\rm{N}}{\rm{u}}{\rm{m}}{\rm{b}}{\rm{e}}{\rm{r}}\,{\rm{o}}{\rm{f}}\,{\rm{e}}{\rm{v}}{\rm{e}}{\rm{n}}{\rm{t}}{\rm{s}}\,{\rm{w}}{\rm{i}}{\rm{t}}{\rm{h}}\,{\rm{s}}{\rm{p}}{\rm{a}}{\rm{c}}{\rm{i}}{\rm{n}}{\rm{g}} < 0}{{\rm{T}}{\rm{o}}{\rm{t}}{\rm{a}}{\rm{l}}\,{\rm{n}}{\rm{u}}{\rm{m}}{\rm{b}}{\rm{e}}{\rm{r}}\,{\rm{o}}{\rm{f}}\,{\rm{c}}{\rm{a}}{\rm{r}}-{\rm{f}}{\rm{o}}{\rm{l}}{\rm{l}}{\rm{o}}{\rm{w}}{\rm{i}}{\rm{n}}{\rm{g}}\,{\rm{e}}{\rm{v}}{\rm{e}}{\rm{n}}{\rm{t}}{\rm{s}}}$$

Like the MSE metric, the collision rate is easy to interpret and provides a standardized way to compare the performance of different algorithms across different datasets.

Jerk refers to the rate of change of acceleration over time and reflects the smoothness of vehicle movements. Lower jerk values indicate smoother acceleration and deceleration, leading to a more comfortable driving experience. The jerk metric can be calculated using the formula:

11 $${\rm{Jerk}}=\frac{{a}_{n}(t)-{a}_{n}(t-1)}{dt}$$

where *a*_*n*_(*t*) and *a*_*n*_(*t*−1) represent the acceleration of the FV at time *t* and time *t*−1, respectively.

Furthermore, TTC represents the time it would take for the two vehicles to collide if they maintained their current states. A higher TTC value indicates a safer following distance between vehicles. The formula for calculating TTC is as follows:

12 $$TTC(t)=-\frac{{S}_{n-1,n}(t)}{\Delta {V}_{n-1,n}(t)}$$

where *S*_*n*−1,*n*_ stands for the spacing distance between the FV and the LV, and Δ*V*_*n*−1,*n*_ is the relative speed (LV’ speed – FV’s speed).

By incorporating these additional metrics, we can better assess the overall performance of car-following models in terms of accuracy, driving comfort, and safety.

## Data Availability

The car-following events extracted from five public driving datasets are available via Figshare^68^.

## Code availability

You can access the car-following data and apply baseline algorithms with trained models, including the parameters of traditional models like IDM and GHR, for each dataset. These datasets and models are publicly available on GitHub at https://github.com/HKUST-DRIVE-AI-LAB/FollowNet.
